# Association of mental disorders with sepsis: a bidirectional Mendelian randomization study

**DOI:** 10.3389/fpubh.2024.1327315

**Published:** 2024-05-17

**Authors:** Yuanzhi Hu, Zihui Xiong, Pinge Huang, Wan He, Minlin Zhong, Danqi Zhang, Guanghua Tang

**Affiliations:** ^1^Guangzhou University of Chinese Medicine, Guangzhou, China; ^2^The Second Clinical Medical College of Guangzhou University of Chinese Medicine, Guangzhou, China; ^3^Emergency Department of Guangdong Provincial Hospital of Traditional Chinese Medicine, Guangzhou, China; ^4^Guangdong Provincial Key Laboratory of Research on Emergency in TCM, Guangzhou, China

**Keywords:** anorexia nervosa, blood metabolites, gut microbiome, inflammatory factors, Mendelian randomization, mental disorders, sepsis

## Abstract

**Background:**

Substantial research evidence supports the correlation between mental disorders and sepsis. Nevertheless, the causal connection between a particular psychological disorder and sepsis remains unclear.

**Methods:**

For investigating the causal relationships between mental disorders and sepsis, genetic variants correlated with mental disorders, including anorexia nervosa (AN), attention-deficit hyperactivity disorder (ADHD), autism spectrum disorder (ASD), bipolar disorder (BD), major depressive disorder (MDD), obsessive-compulsive disorder (OCD), panic disorder (PD), posttraumatic stress disorder (PTSD), schizophrenia (SCZ), and tourette syndrome (TS), were all extracted from the Psychiatric Genomics Consortium (PGC). The causal estimates and direction between these mental disorders and sepsis were evaluated employing a two-sample bidirectional MR strategy. The inverse variance weighted (IVW) method was the primary approach utilized. Various sensitivity analyses were performed to confirm the validity of the causal effect. Meta-analysis, multivariable MR, and mediation MR were conducted to ensure the credibility and depth of this research.

**Results:**

The presence of AN was in relation to a greater likelihood of sepsis (OR 1.08, 95% CI 1.02–1.14; *p =* 0.013). A meta-analysis including validation cohorts supported this observation (OR 1.06, 95% CI 1.02–1.09). None of the investigated mental disorders appeared to be impacted when sepsis was set as the exposure factor. Even after adjusting for confounding factors, AN remained statistically significant (OR 1.08, 95% CI 1.02–1.15; *p =* 0.013). Mediation analysis indicated N-formylmethionine levels (with a mediated proportion of 7.47%), cystatin D levels (2.97%), ketogluconate Metabolism (17.41%) and N10-formyl-tetrahydrofolate biosynthesis (20.06%) might serve as mediators in the pathogenesis of AN-sepsis.

**Conclusion:**

At the gene prediction level, two-sample bidirectional MR analysis revealed that mental disorder AN had a causal association with an increased likelihood of sepsis. In addition, N-formylmethionine levels, cystatin D levels, ketogluconate metabolism and N10-formyl-tetrahydrofolate biosynthesis may function as potential mediators in the pathophysiology of AN-sepsis. Our research may contribute to the investigation of novel therapeutic strategies for mental illness and sepsis.

## 1 Introduction

As a common syndrome in emergency departments and intensive care units (ICUs), sepsis is a potentially fatal organ dysfunction attributed to a dysregulated host reaction to infection (1). In 2017, it harmed 48.9 million individuals globally, with 11 million fatalities in relation to sepsis, representing approximately 20% of overall mortality worldwide (2), and remains one of the primary factors of patient mortality globally. In recent years, although the age standardized incidence rate and mortality of sepsis have declined worldwide, many sepsis survivors suffer from cognitive decline, mental health disorders, physical function damage, and long-term death risks (3, 4). Severe sepsis and septic shock increase the likelihood of psychiatric disorders including major depressive disorder (MDD) (5), anxiety disorder (AD) and posttraumatic stress disorder (PTSD) (6).

Mental disorders pose a serious threat to public health because they serve as a critical cause of numerous diseases. Previous studies have indicated that mental disorders are the primary risk factors related to the advancement of sepsis. For instance, retrospective multicenter research revealed that patients previously diagnosed with mental illnesses, including MDD, attention deficit hyperactivity disorder (ADHD), schizophrenia (SCZ), bipolar disorder (BD) and panic disorder (PD), possess a greater likelihood of severe sepsis and related death (7). However, a nationwide cohort study from France suggested that septic patients with BD, MDD, and SCZ, have lower 90-day case fatality rates than other individuals (8). These inconsistent research results are contradictory, and the assessment of the types of mental disorders is not comprehensive, which limits the opportunities for guiding early prevention and effective treatment of diseases.

Mendelian randomization (MR) serves as an epidemiological approach that utilizes instrumental variable single nucleotide polymorphisms (SNPs) for random allocation (9). SNPs are highly associated with exposure factors, reducing the susceptibility of MR analysis to reverse causality bias and confounding factors, independent of potential confounding factors (10). Consequently, the statistical efficacy of causal connections may be increased by analyzing summary data from GWAS applying two-sample MR (11). Multivariable MR (MVMR) is an advanced approach that extends MR by utilizing genetic variants linked to multiple possibly interconnected exposures to assess the impact of each exposure on a single outcome. This approach could avoid bias induced by confounding factors, preserving the advantages of utilizing genetic instruments for causal inference (12). The association between sepsis and different illnesses, such as insomnia (13), type I diabetes mellitus (14), atrial fibrillation, and cardioembolic stroke (15), has been extensively investigated utilizing MR analysis. Nevertheless, research has concentrated less on the relationship between sepsis and mental disorders.

Consequently, a two-sample bidirectional MR analysis was conducted utilizing the aggregated statistical data from the Large Genome Association Study (GWAS) to evaluate the causal association of 12 mental disorders, including ADHD, BD, MDD, PD, PTSD, SCZ, anorexia nervosa (AN), tourette syndrome (TS), obsessive-compulsive disorder (OCD), and autism spectrum disorder (ASD), on the risk of sepsis. Additionally, the causal involvement of sepsis in the onset of these 10 psychiatric disorders is also examined. Meta-analyses were performed to prove the credibility of the causal associations utilizing the validation cohorts. Based on previous studies, total cholesterol, triglyceride levels, and weight were recognized as possible confounding factors (16–22), and MVMR was carried out to ensure the independent causal impact of mental disorders on sepsis. Additionally, we explored the potential mediators between mental illness and sepsis. Previous studies have shown that mental illness leads to changes in inflammatory factors, blood metabolites and gut microbiome, which in turn are associated with sepsis (23–28), and several MR studies have recently demonstrated the relationship between blood metabolites and sepsis (29, 30). Therefore, mediation MR analysis was applied to explore the mediating pathway among mental illnesses and sepsis via the phenotypes of inflammatory factors, blood metabolites and gut microbiome.

## 2 Methods

### 2.1 Study design

**Figure 1:**
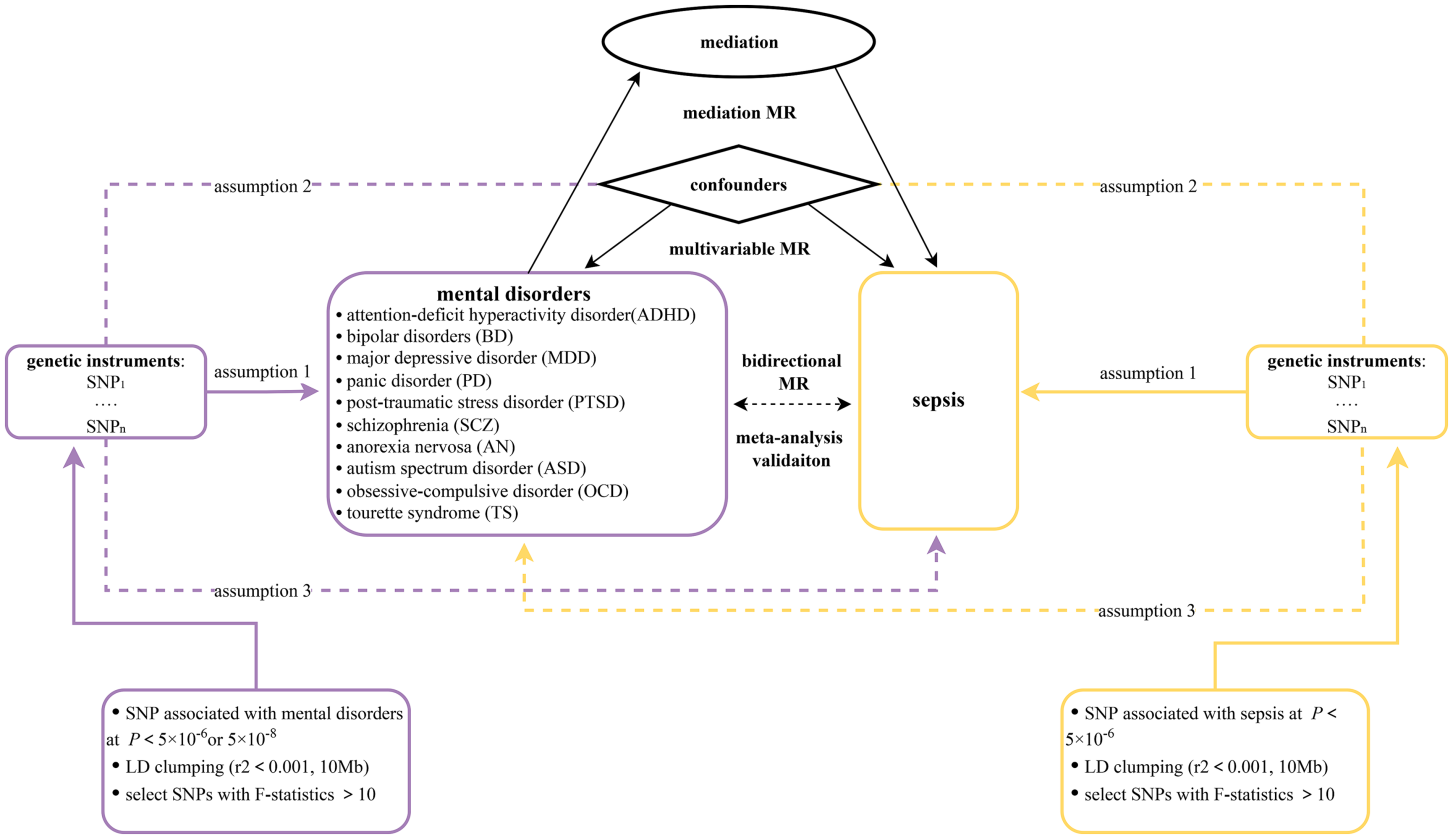
Flowchart overview of the Mendelian randomization study.

This study is reported following the Strengthening the Reporting of Observational Studies in Epidemiology Using Mendelian Randomization guidelines (STROBE-MR, Supplementary Table S1) (31). In this research, a two-sample bidirectional MR design was applied to claim the causal effect between 10 mental disorders (ADHD, BD, MDD, PD, PTSD, SCZ, AN, TS, OCD, and ASD) and sepsis. First, we assumed 10 mental disorders as exposures and sepsis as outcome. To establish a causal connection between mental illnesses and sepsis, forward MR studies were performed. Second, the effect of sepsis on 10 mental illnesses was examined utilizing reverse MR analyses. MR analyses must meet three basic assumptions: (1) genetic variants are strongly correlated with exposures; (2) genetic variants are not linked to any confounders; and (3) genetic variants only influence outcomes only through exposure (32). The most recent GWAS datasets from the FinnGen (33) database were combined with the latest study published by Hamilton et al. (34). The reliability and stability of the causal connection between mental illnesses and sepsis were assessed using a meta-analysis methodology. The MVMR method was employed in order to reduce potential pleiotropy by adjusting for confounding variables including total cholesterol, triglyceride levels, and weight. In the mediation analysis, 91 inflammatory factors, 309 metabolite ratios, 1,091 blood metabolites, 205 gut microbiome pathways and 207 gut microbiome taxa were included. The mediation MR approach was utilized to evaluate the indirect effect of each mediator (35). Figure 1 displays the flow chart illustrating the detailed process of our MR analysis. To decrease discrimination based on population stratification, all summarized GWAS data were restricted to participants of European ancestry.

### 2.2 GWAS data sources

The summary statistics for ADHD (36) included data from 13 cohorts, with an amount of 225,534 individuals. Among these individuals, there were 38,691 cases and 186,843 controls. Notably, 49.61% of the participants were female. The definition of ADHD was determined by using the criteria of the International Classification of Diseases, Tenth Revision (ICD-10) code or through clinical assessment, including the consumption of medication specially recommended for the treatment of ADHD. The summary statistics for BD (37) were obtained from a group of 57 cohorts containing 41,917 cases and 371,549 controls. Regarding MDD (38), a comprehensive dataset containing 135,458 cases and 344,901 controls was gathered from 35 various cohorts. The latest GWAS meta-analysis of 6 cohorts with an overall sample size of 2,248 cases and 7,992 controls, provided PD (39) summary statistics. For SCZ (40), summary statistics were collected from 76 cohorts involving a general of 55,365 cases and 21,9,884 controls. The cases diagnosed with BD, MDD, PD, or SCZ were classified based on the Diagnostic and Statistical Manual of Mental Disorders-IV (DSM-IV), ICD-9, or ICD-10 code, and assessed by trained interviewers, clinician-managed checklists, and records of medical review. PGC-PTSD, which comprised 9 European ancestry datasets with 2,424 cases and 7,113 controls, provided the summary statistics for PTSD (41). The diagnostic criteria involved PTSD with the presence of clinical characteristics or self-reported. AN (42) summary statistics were derived from 12 cohorts, totaling 3,495 cases and 10,982 controls. The diagnosis of AN included enduring anorexia nervosa, or long-term eating disorders. Regarding ASD (43), the summary statistics for ASD encompassed a collective count of 18,381 cases and 27,969 controls. Patients with ASD were identified based on the ICD-10 codes. Summary statistics for OCD (44) comprised a sample size of 2,688 patients and 7,037 controls. All cases satisfied the diagnostic criteria for OCD as specified in the DSM-IV. Summary statistics for TS (45) included 4,819 cases and 9,488 controls. The diagnoses of TS cases were established by highly qualified clinicians following DSM-IV-TR criteria. All data were restricted to individuals with European ancestry so as to prevent potential bias induced by population heterogeneity. We manually checked all cohorts of GWAS meta-analytic results for 10 mental disorders and discarded those results containing UK Biobank participants, which could largely minimize sample overlap and ensure independence between the exposure and outcome data.

Summarized GWAS data for sepsis were derived from the UK Biobank (46), comprising 462,918 individuals with 10,154 cases and 454,764 controls of European ancestry. The BOLT-LMM linear mixed model was applied to adjust the genetic association estimates for sepsis based on factors such as age, sex, and genotyping chip. The diagnosis of sepsis was primarily determined based on a structured assessment by a panel of physicians. An earlier published paper offered more specific information about this summarized GWAS data (47). The cohort of sepsis cases comprised 5,655 females (56%), with a median age of 60 years, a mean BMI of 28.2 kg/m^2^, mean low-density lipoprotein cholesterol levels of 3.4 mmol/L, and a mean systolic blood pressure of 136.1 mmHg. These baseline characteristics for sepsis cases were consistent with those observed in the comparator groups. Furthermore, we selected two datasets for validation to improve the stability of the causal association between mental illness and sepsis to a greater extent. We collected GWAS data on sepsis from a study conducted by Hamilton et al. (34). The sample comprised 486,484 individuals of European ancestry, of which 11,643 were cases and 474,841 were controls. The FinnGen R10 database was used as another validation source, the data on sepsis from which included 429,209 European-ancestry individuals with 13,531 cases and 415,678 controls (33). All the sepsis cases mentioned above had to match the criteria including the ICD-10 codes A02, A39, A40, and A41. All GWAS data were derived from individuals with European genetic ancestry. And individuals with sex mismatches, genotype missingness greater than 5 per cent and heterozygosity greater than or less than four standard deviations were excluded (33). And it is worth noting that FinnGen R10 database contains only individuals of Finnish genetic ancestry.

Summary statistics for total cholesterol levels were gathered from Nightingale Health’s metabolomic research and included a sample size of 115,078 patients. The summary data for triglyceride levels were gathered from the Within Family GWAS Consortium, which included 30,515 individuals. Both datasets were downloaded from the IEU OpenGWAS database.^1^ Summary statistics for weight were derived from the FinnGen database and included a sample size of 1,146 cases and 217,646 controls (33). GWAS data for 91 inflammatory factors were derived from 11 cohorts comprising a total of 14,824 individuals with European ancestry (48). Summary statistics for 309 metabolite ratios and 1,091 blood metabolites were gathered from The Canadian Longitudinal Study on Aging for 51,338 randomly selected participants aged 45–85 years (49). Summary statistics for 205 pathways and 207 taxa in relation to gut microbiome were acquired from the Dutch Microbiome Project, analyzing feces from 7,738 individuals (50). The use of data sources distinct from the outcome data was confirmed to avoid sample overlap. Further information regarding summarized GWAS data related to exposure and outcome is provided in Table 1. All original studies were ethically approved, and all original data used in this study were openly accessible.

**Table 1:** Summary of genome-wide association studies (GWAS) datasets for 10 mental disorders and sepsis.

Traits	Data sources	Years	Ancestry	Sample size (cases/controls)	WebSource/PubMed ID
BD	PGC, iPSYCH, deCODE, Estonian Biobank, HUNT	2021	European	413,466 (41,917/371,549)	36,702,997
MDD	PGC29, deCODE, GenScotland, GERA, iPSYCH, 23andMeD	2018	European	480,359 (135,458/344,901)	34,002,096
SCZ	PGC, deCODE	2022	European	275,249 (55,365/219,884)	35,396,580
AN	CHOP/PFCG, GCAN/WTCCC3	2018	European	14,477 (3,495/10,982)	31,308,545
ASD	iPSYCH, PGC	2019	European	46,350 (18,381/27,969)	30,804,558
OCD	IOCDF-GC, OCGAS	2018	European	9,725 (2,688/7,037)	28,761,083
ADHD	iPSYCH, deCODE, PGC	2023	European	225,534 (38,691/186,843)	36,702,997
PTSD	PGC-PTSD	2018	European	9,537 (2,424/7,113)	31,594,949
TS	TAAICG, PGC-TS, TIC, deCODE	2019	European	14,307 (4,819/9,488)	30,818,990
PD	Germany I, Germany II, Germany III, Sweden, Denmark, Estonia	2021	European	10,240 (2,248/7,992)	31,712,720
Sepsis (main analysis)	UK Biobank	2020	European	462,918 (10,154/454,764)	32,966,752
Sepsis (validation)	Hamilton F et al.	2023	European	486,484 (11,643/474,841)	36,716,318
Sepsis (validation)	FinnGen	2023	European	429,209 (13,531/415,678)	36,653,562
Total cholesterol levels	Nightingale Health’s metabolomic research	2020	European	115,078	https://gwas.mrcieu.ac.uk/
Triglyceride levels	Within Family GWAS Consortium	2022	European	30,515	https://gwas.mrcieu.ac.uk/
Weight	FinnGen	2021	European	218,792 (1,146/217,646)	36,653,562
Inflammatory factors	Zhao JH et al.	2023	European	14,824	37,563,310
Blood metabolites	The Canadian Longitudinal Study	2023	European	51,338	36,635,386
Gut microbiome	Dutch Microbiome Project	2022	European	7,738	35,115,690

BD, bipolar disorders; MDD, major depressive disorder; SCZ, schizophrenia; AN, anorexia nervosa; ASD, autism spectrum disorder; OCD, obsessive-compulsive disorder; ADHD, attention-deficit hyperactivity disorder; PTSD, posttraumatic stress disorder; TS, tourette syndrome; PD, panic disorder; PGC, Psychiatric Genomics Consortium.

### 2.3 Selection of instrument variants

As stated previously, SNPs for exposure were selected with a stringent *p*-value criteria (*p* < 5 × 10^−8^). However, few independent SNPs were identified by these strict criteria. In light of this situation, we applied a less restrictive statistical threshold (*p* < 5 × 10^−6^) for the exposure variables (MDD, AN, PD, PTSD, ASD, OCD, TS, sepsis, total cholesterol, triglyceride levels, weight). Moreover, a higher cutoff (*p* < 1 × 10^−5^) was selected considering the relatively few SNPs discovered for part of mediators when they served as exposures. This strategy has been applied in several MR studies (51, 52). Then, we specified a clumping window of 10,000 kb and linkage disequilibrium *R*^2^ < 0.001 to guarantee the independence of the selected SNPs (53). Furthermore, we manually checked and removed genetic variants that were not identified in the outcome GWAS data to guarantee that the genetic variants included could directly link psychiatric disorders to sepsis. Second, SNPs that had a strong correlation with the outcome were excluded. Second, F-statistics (F = beta^2^/se^2^) were calculated for each SNP (54). In an effort to minimize the impact for weak instrumental bias, SNPs were ruled out if their corresponding F-statistics failed to reach the threshold of 10. Third, harmonization of effect estimates was performed to verify that exposure data and outcome data had corresponding effect directions and to avoid strand mismatch (55, 56).

### 2.4 Statistical analysis

The principal method employed in the major analysis was IVW analysis, which was chosen for its strong statistical power (57). The IVW approach combined the Wald ratios derived from each SNP, assuming SNPs with the lowest variance. For binary outcomes, the measures of causal estimates are presented as the form of odds ratio (OR) and 95% confidence interval (CI). Due to the estimation method of IVW was unbiased, it is susceptible to invalid instrument bias (13). As a result, additional complementary methods such as maximum likelihood (ML), weighted median (WM), and MR-Egger were conducted to assure the validity of the results. To facilitate the interpretation of the intercepts and directions of the different analysis methods, scatter plots were generated. Moreover, the Cochrane’s Q test for the IVW method and funnel plot were utilized to determine heterogeneity (58). The *p*-value of Cochran’s Q test was also applied in choosing random-effects (*p* < 0.05) or fixed-effects IVW MR analysis (*p* > 0.05). Subsequently, estimates of horizontal pleiotropy were derived using the MR-Egger intercept tests (59). Last but not least, leave-one-out analysis was applied to assess the potential presence of bias in the estimations due to the influence of a single SNP.

The GWAS datasets from distinct sources were merged via meta-analysis method to assess the stability and credibility of the previous results. By adjusting for confounding risk factors with MVMR analysis, we were able to reduce the impact of confounding variables on causal associations. Applying instrumental variables for mental diseases, we first assessed the causal influence of mental illnesses on a hypothesized mediator. In the following phase, we established the causal impact of the mediators on sepsis by utilizing instrumental variables as the mediator. We quantified the mediation proportion of each mediating component by dividing the indirect effect by the total effect. The delta approach was used to estimate confidence intervals (60). Sensitivity analyses were applied to prove the validity of the results. The entire data preparation and analyses were conducted utilizing the TwoSampleMR (version 0.5.7), MendelianRandomization (version 0.9.0), and meta (version 7.0-0) packages in the R software (version 4.3.0).

## 3 Results

### 3.1 Genetic instrumental variables

We conducted a comprehensive screening of instrumental variables for 10 psychiatric disorders, and all 294 SNPs for psychiatric disorders associated with sepsis were ultimately included in the research. There were between 8 and 122 SNPs for each psychiatric disorder. Additionally, instrumental variables for sepsis were comprised of 32 SNPs, with 12 SNPs being utilized in the validation cohorts. There were between 10 and 111 SNPs for each confounding factor. The number of SNPs utilized in mediation MR varies from 7 to 45. The F-statistics exceeding 10 were observed for each of the instrumental variables. Supplementary Tables S2–S5 provide comprehensive details on the chosen instrumental variables and the harmonized data.

### 3.2 Bidirectional MR analysis

**Figure 2:**
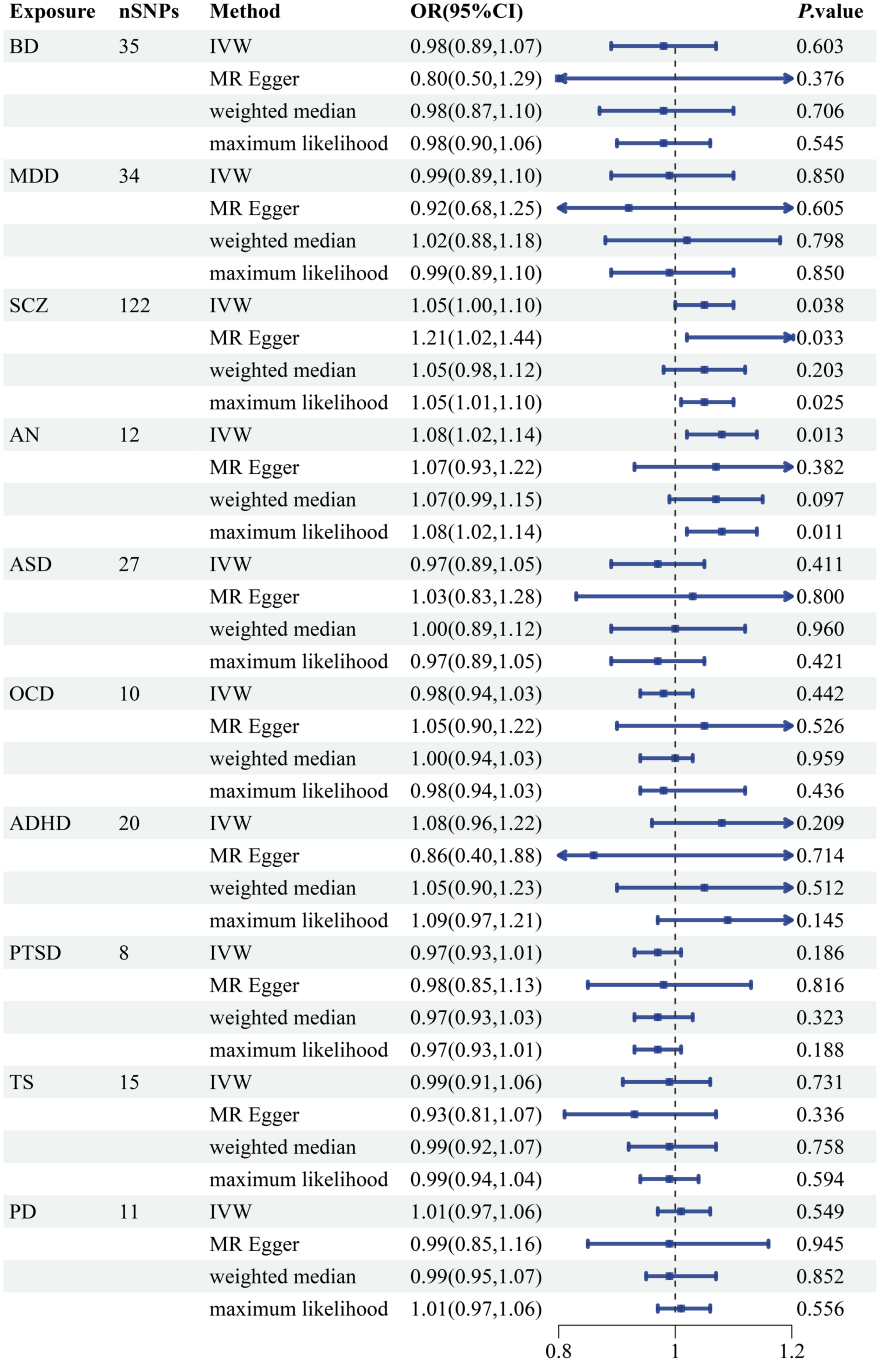
Forest plot of causal associations between 10 mental disorders and sepsis risk. BD, bipolar disorder; MDD, major depressive disorder; SCZ, schizophrenia; AN, anorexia nervosa; ASD, autism spectrum disorder; OCD, obsessive-compulsive disorder; ADHD, attention-deficit hyperactivity disorder; PTSD, posttraumatic stress disorder; TS, tourette syndrome; PD, panic disorder; SNP, single nucleotide polymorphism; IVW, inverse variance weighted; OR, odds ratios; CI, confidence intervals; *p*-value < 0.05 was considered significant.

**Figure 3:**
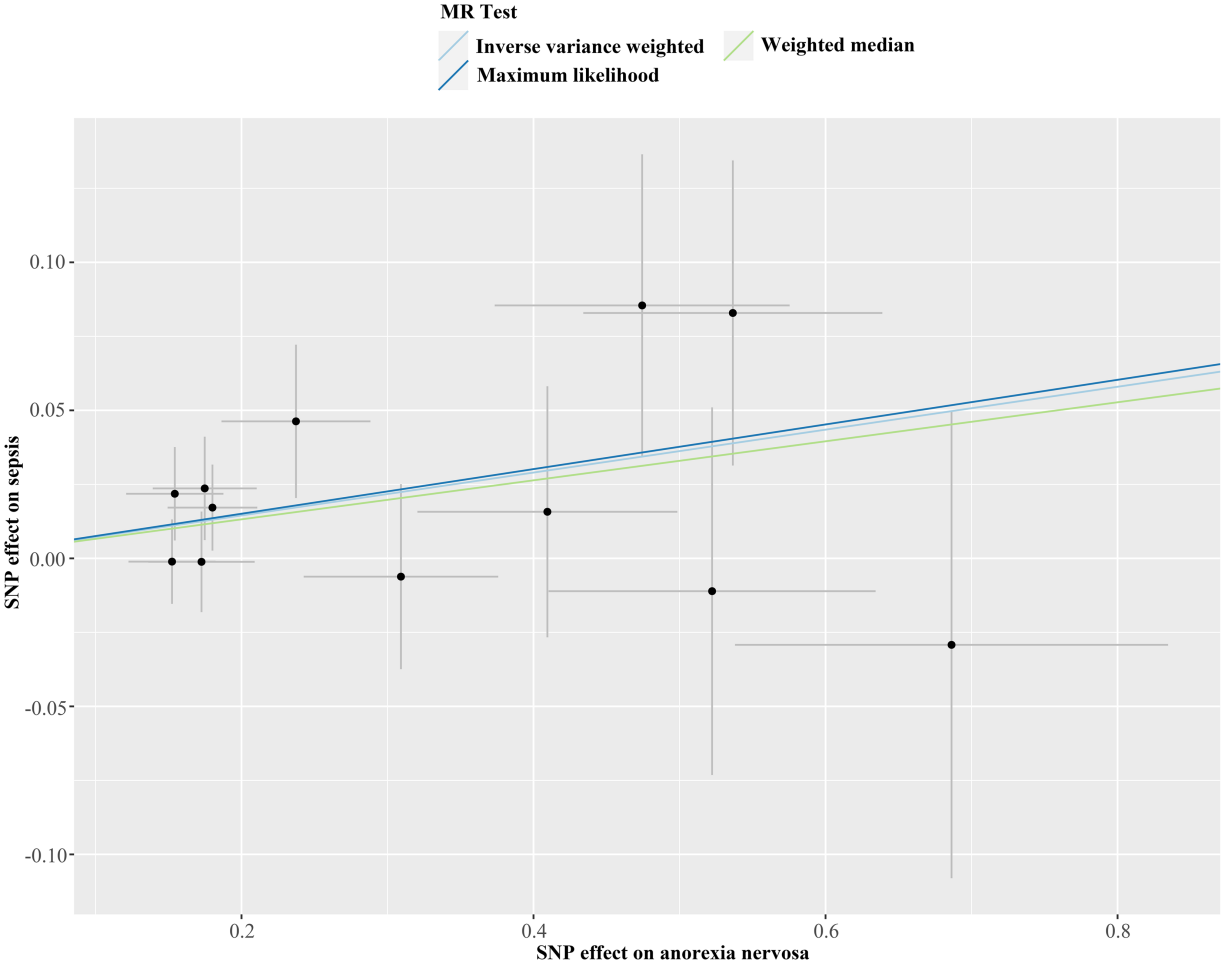
Scatter plot of Mendelian randomization analysis for the associations of anorexia nervosa with the risk of sepsis. SNP, single nucleotide polymorphism; MR, Mendelian randomization.

**Figure 4:**
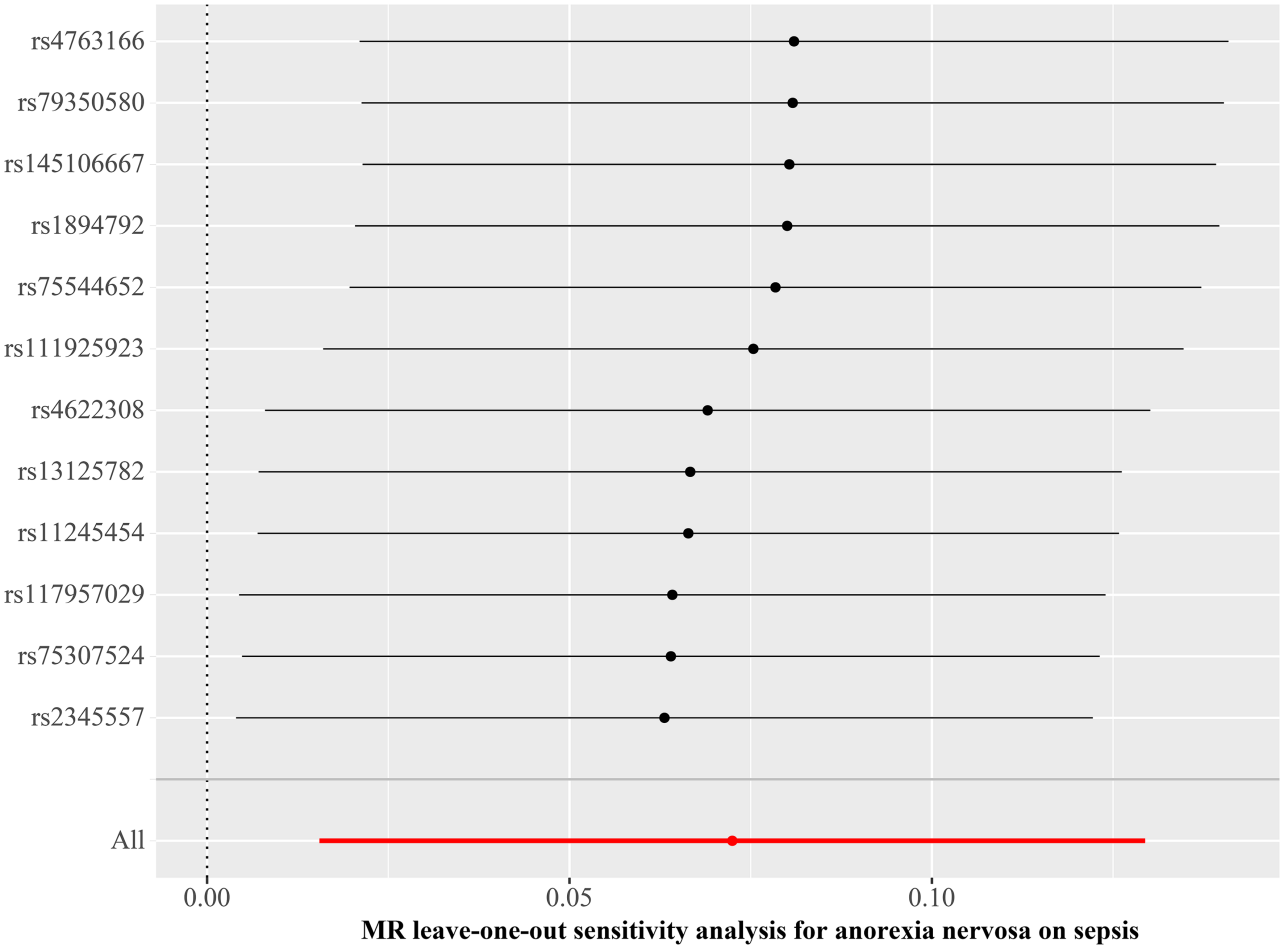
Leave-one-out plot for Mendelian randomization analysis of the causal effect of anorexia nervosa on the risk of sepsis. MR, Mendelian randomization.

We discovered a correlation between sepsis and genetic susceptibilities to AN by using mental diseases as exposures to examine the causal effect of mental diseases on sepsis. The IVW analysis revealed a correlation between AN and an elevated risk of sepsis (OR 1.08, 95% CI 1.02–1.14; *p =* 0.013). ML analysis provided the same estimate (OR 1.08, 95% CI 1.02–1.14; *p =* 0.010), while MR-Egger and WM analyses yielded consistent yet nonsignificant results (Figure 2). The scatter plot demonstrated that the analysis results were nearly identical direction (Figure 3). Using mental disorders as the outcomes, the *p*-values of all 10 IVW analyses were greater than 0.05, indicating that sepsis failed to affect any of the studied mental disorders (Table 2; Supplementary Table S6). A variety of sensitivity analysis approaches, such as the Cochran’s Q test, the MR Egger intercept, the funnel plot, and the leave-one-out analysis, were used to evaluate the reliability of the positive estimates. The Cochran’s Q test performed for AN as an exposure factor showed *p*-values larger than 0.05, and the *p*-value for the MR Egger intercept was found to exceed the predetermined threshold of 0.05, suggesting no evidence of heterogeneity and horizontal pleiotropy in our study (Table 3). In addition, the funnel plot was symmetric, further demonstrating the validity of the results (Supplementary Figure S1). Finally, using leave-one-out analysis to avoid biasing effects on IVW, the correlation between AN and a higher likelihood of sepsis was meticulously confirmed (Figure 4).

**Table 2:** Results of reverse MR analyses and sensitivity analysis assumed sepsis as exposure and 10 mental disorders as outcome.

Exposure	Outcome	nSNPs	IVW		Cochran Q test		MR-Egger intercept tests	
			OR (95%CI)	*p*	*Q* value	*p*	MR-Egger intercept	*p*
Sepsis	BD	18	0.96 (0.87, 1.05)	0.32	30.305	0.024	−0.005	0.712
	MDD	22	0.92 (0.76, 1.11)	0.37	16.147	0.761	0.010	0.698
	SCZ	20	0.95 (0.86, 1.05)	0.32	21.409	0.315	−0.014	0.322
	AN	18	0.95 (0.89, 1.02)	0.18	12.416	0.774	−0.011	0.305
	ASD	24	1.01 (0.95, 1.07)	0.75	17.530	0.782	−0.002	0.765
	OCD	20	1.20 (0.93, 1.55)	0.15	24.280	0.186	0.014	0.693
	ADHD	24	1.25 (0.97, 1.61)	0.08	25.200	0.340	0.004	0.906
	PTSD	24	1.12 (0.87, 1.44)	0.37	22.682	0.479	−0.026	0.439
	TS	24	0.97 (0.92, 1.03)	0.31	21.278	0.564	−0.007	0.330
	PD	20	1.12 (0.93, 1.35)	0.23	21.098	0.331	0.023	0.370

BD, bipolar disorders; MDD, major depressive disorder; SCZ, schizophrenia; AN, anorexia nervosa; ASD, autism spectrum disorder; OCD, obsessive-compulsive disorder; ADHD, attention-deficit hyperactivity disorder; PTSD, posttraumatic stress disorder; TS, tourette syndrome; PD, panic disorder; IVW, inverse variance weighted; *p* < 0.05 was considered significant.

**Table 3:** Sensitivity analysis of the associations between 10 mental disorders and sepsis.

Exposure	Outcome	Cochran Q test		MR-Egger intercept tests	
		*Q* value	*p*	MR-Egger intercept	*p*
BD	Sepsis	44.584	0.106	0.013	0.421
MDD		30.578	0.588	0.005	0.630
SCZ		139.240	0.123	−0.010	0.098
AN		7.864	0.725	0.002	0.903
ASD		26.320	0.446	−0.006	0.557
OCD		8.169	0.517	−0.019	0.372
ADHD		24.492	0.178	0.014	0.571
PTSD		2.568	0.922	−0.008	0.881
TS		30.460	0.007	0.014	0.350
PD		7.088	0.717	0.010	0.795

BD, bipolar disorders; MDD, major depressive disorder; SCZ, schizophrenia; AN, anorexia nervosa; ASD, autism spectrum disorder; OCD, obsessive-compulsive disorder; ADHD, attention-deficit hyperactivity disorder; PTSD, posttraumatic stress disorder; TS, tourette syndrome; PD, panic disorder; *p* < 0.05 was considered significant.

### 3.3 Meta-analysis

**Figure 5:**
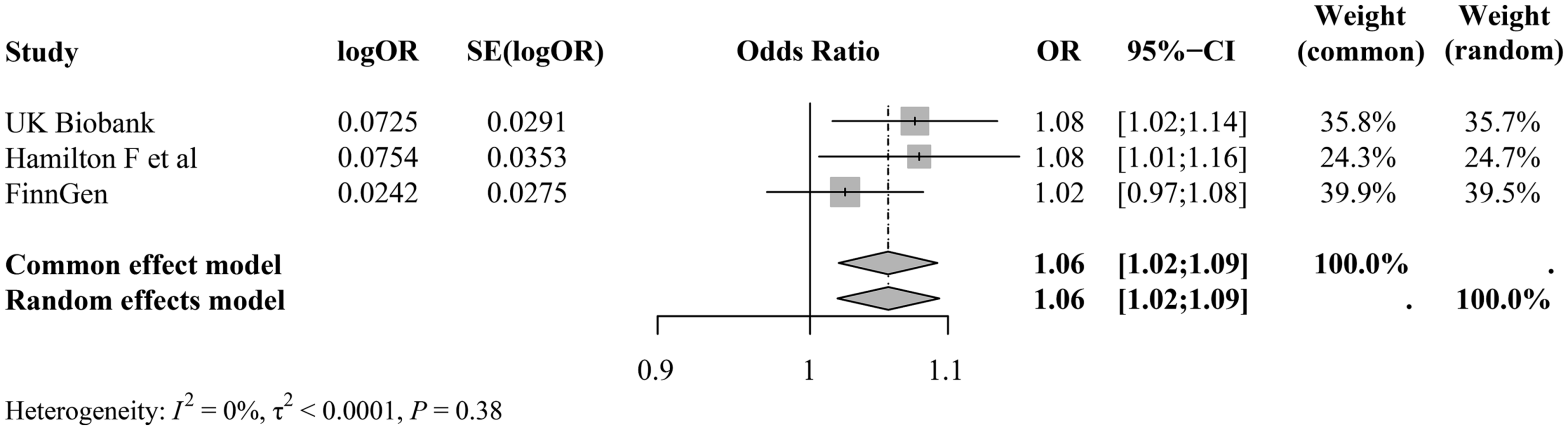
Meta-analysis of the causal effect of AN on sepsis. SE, standard error; OR, odds ratio; CI, confidence interval; *p* < 0.05 was considered significant.

As demonstrated in Figure 5, the ORs of the three datasets were in identical direction, validating the effect of AN on the increased risk of sepsis occurrence, and with the inclusion of the two validation datasets, the effect of AN on the increased possibility of sepsis occurrence (OR 1.08, 95% CI 1.02–1.14) remained relatively stable in the meta-analysis model (OR 1.06, 95% CI 1.02–1.09) and no heterogeneity was observed (*I*^2^ = 0%, τ^2^ < 0.0001, *p =* 0.38).

### 3.4 MVMR

**Figure 6:**
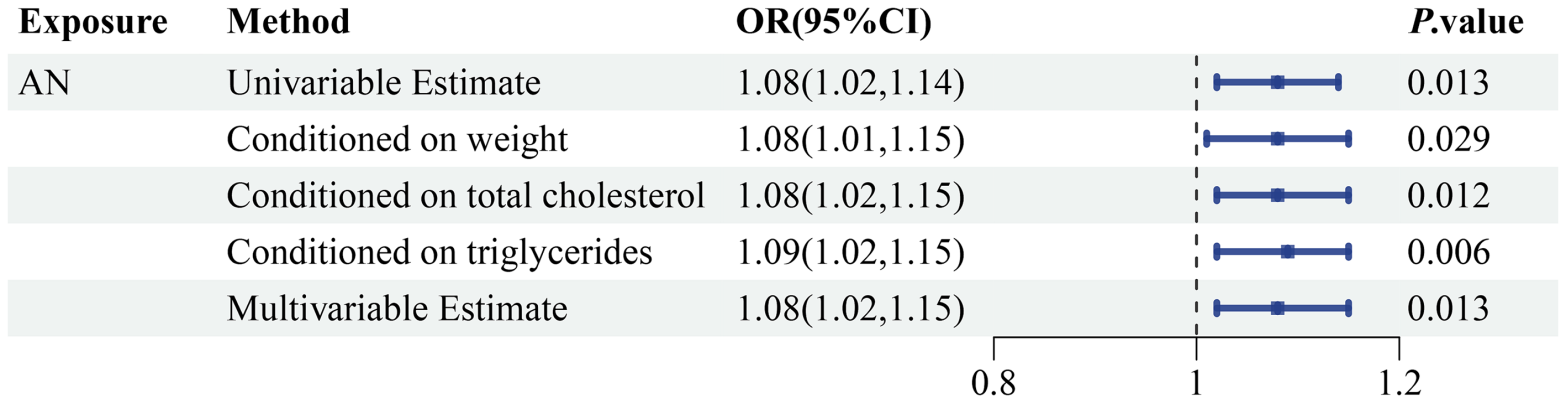
Genetically predicted association of AN with sepsis after adjusting for confounding factors. OR, odds ratio; CI, confidence interval; *p*-value < 0.05 was considered significant.

In order to more thoroughly exclude the impact of confounding variable-level pleiotropy, we employed the MVMR method. An association of statistical significance remained between AN and sepsis after adjusting for total cholesterol (OR 1.08, 95% CI 1.02–1.15; *p =* 0.012), triglyceride levels (OR 1.09, 95% CI 1.02–1.15; *p =* 0.006), and weight (OR 1.08, 95% CI 1.01–1.15; *p =* 0.029). Moreover, we included all the confounding factors to conduct a multivariable estimate; the investigation remained stable and confirmed previous results (OR 1.08, 95% CI 1.02–1.15; *p =* 0.013) (Figure 6; Supplementary Table S7). The Cochran’s Q test conducted for AN as an exposure factor indicated *p*-values greater than 0.05. The *p*-value for the MR Egger intercept in all analyses was more than the predefined threshold of 0.05 (Supplementary Table S8). Our investigation failed to identify any evidence of heterogeneity or horizontal pleiotropy.

### 3.5 Mediation MR

**Figure 7:**
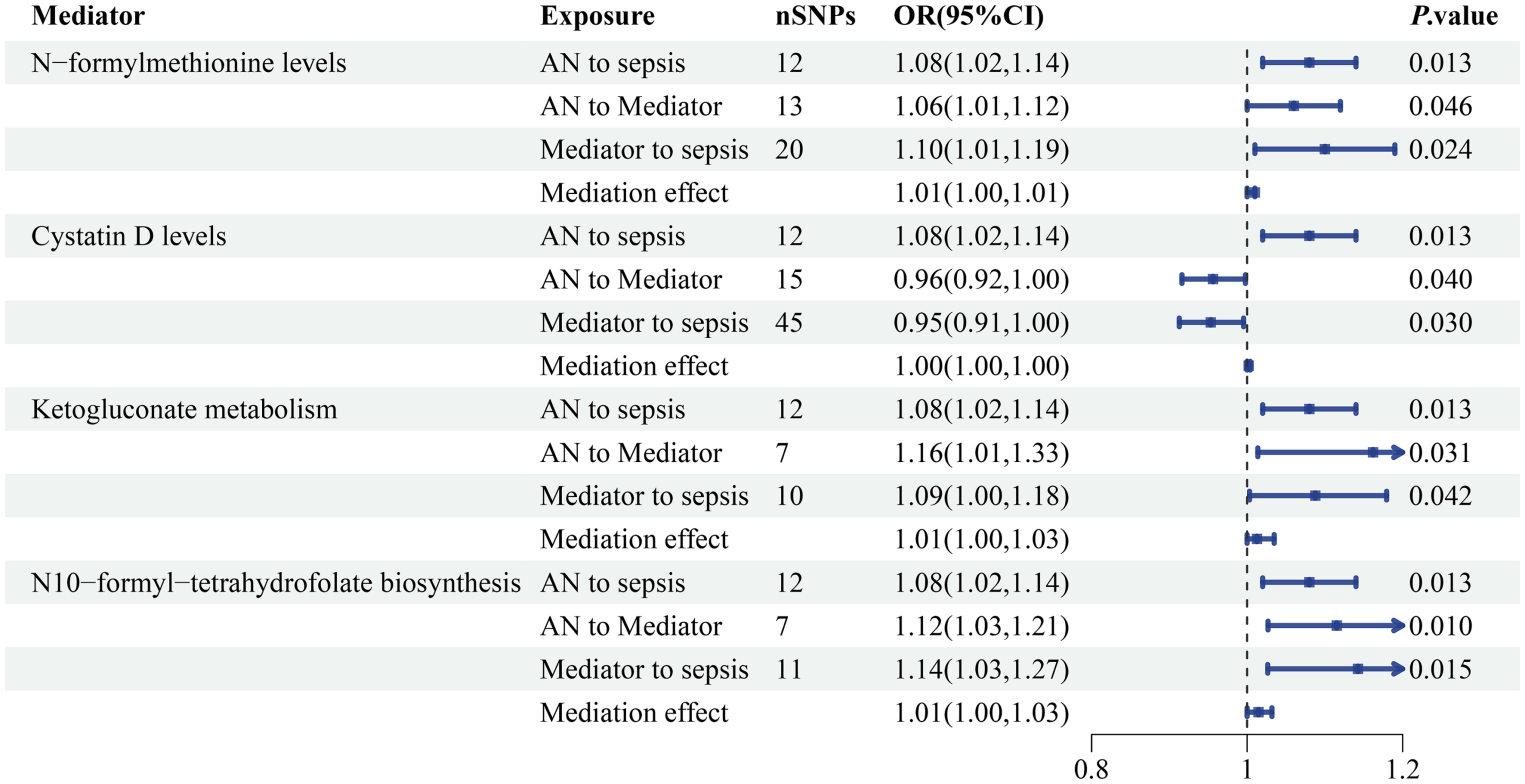
The mediating effect of AN on sepsis via blood N-formylmethionine levels and cystatin D levels accompanied by ketogluconate metabolism and N10-formyl-tetrahydrofolate biosynthesis in the gut microbiome. SNP, single nucleotide polymorphism; OR, odds ratio; CI, confidence interval; *p*-value < 0.05 was considered to indicate statistical significance.

Among the 91 inflammatory factors, 309 metabolite ratios, 1,091 blood metabolites, 205 gut microbiome pathways and 207 gut microbiome taxa examined, N-formylmethionine levels, cystatin D levels, ketogluconate metabolism, and N10-formyl-tetrahydrofolate biosynthesis were found to be associated with the causal association between AN and sepsis. The causative influence of AN on N-formylmethionine levels was statistically significant (OR 1.06, 95% CI 1.01–1.12; *p =* 0.046). Also in the causative influence of AN on cystatin D levels (OR 0.96, 95% CI 0.92–1.00; *p =* 0.040), ketogluconate metabolism (OR 1.16, 95% CI 1.01–1.33; *p =* 0.031) and N10-formyl-tetrahydrofolate biosynthesis (OR 1.12, 95% CI 1.03–1.21; *p =* 0.010). This research revealed a causal association between N-formylmethionine levels (OR 1.10, 95% CI 1.01–1.19; *p =* 0.024), cystatin D levels (OR 0.95, 95% CI 0.91–1.00; *p =* 0.030), ketogluconate metabolism (OR 1.09, 95% CI 1.00–1.18; *p =* 0.042), N10-formyl-tetrahydrofolate biosynthesis (OR 1.14, 95% CI 1.03–1.27; *p =* 0.015) and sepsis (Figure 7). In conjunction with previous studies establishing a causal relationship between AN and sepsis, a mediation analysis was performed. The mediation analysis revealed that N-formylmethionine levels mediated the connection between AN and sepsis and explained 7.47% of the mediation impact. Cystatin D levels (2.97%), ketogluconate metabolism (17.41%), and N10-formyl-tetrahydrofolate biosynthesis (20.06%) were additionally included. No heterogeneity or pleiotropy was detected. The detailed results and sensitivity analysis of the mediation MR can be found in Supplementary Tables S9–S11.

## 4 Discussion

This study presented genetic data suggesting a causal connection between mental diseases and sepsis through bidirectional MR, meta-analysis, and MVMR analyses. Mediators between mental illness and sepsis were investigated by mediation MR analysis.

Clinical investigations have indicated a connection between mental disorders and sepsis. Large prospective cohort studies revealed that MDD and PD were linked to a higher likelihood of sepsis (6). Additionally, MDD, PD, and PTSD were observed at increased rates among individuals with sepsis (61). A clinical investigation revealed that MDD is in relation to increased 5-year all-cause mortality in individuals who survived sepsis (62). Nevertheless, a nationwide cohort study conducted in France indicated that septic patients with severe mental illnesses (BD, MDD, and SCZ) have reduced 90-day case fatality rates (8). Several research focused on the correlation between mental illness and infection by employing the Mendelian randomization method. The inflammatory factors CRP and IL-6R were found to be associated with SCZ (63). MDD and ADHD elevate the likelihood of hospitalization due to COVID-19, whereas SCZ is also strongly associated with COVID-19 (64–66). Clinical studies on the correlation between mental disorders and sepsis have not been thorough or comprehensive. There are contradictory findings in this research area. In addition, the Mendelian randomization method for establishing a causal association between mental disorders and sepsis is lacking. In order to surmount these obstacles, A MR study was implemented with the purpose of establishing a causal relationship between mental illnesses and sepsis, thereby presenting significant evidence. Our findings revealed compelling evidence that AN was correlated with a higher probability of sepsis. There is no study suggested that sepsis affects any of the studied mental disorders.

AN is a serious psychiatric disease characterized by an elevated death rate, surpassing that of all other psychiatric conditions (67). And this condition is strongly linked to several psychiatric disorders including MDD, OCD, PTSD, and substance abuse disorders (68). In contrast to other mental illnesses, its characteristics mainly include a restriction of calorie consumption relative to requirements, an intense dread of gaining weight or of turning obese, and an irrational preoccupation with shape or body weight (69). The combination of these factors will lead to a series of symptoms such as starvation, malnutrition, and weight loss (70). Research indicates that starvation and malnutrition are key factors contributing to the impairment of the human immune system, potentially resulting in a considerable decline in cellular immunity (71). Metabolic changes in AN could result in significant biochemical disorders, leading to dysfunction in multiple organs and systems, with cytokines and immune cells playing a crucial role. Severe malnutrition causes disturbances in T-cell populations, leading to a higher vulnerability to infections (72). Clinical research on individuals with AN observed decreased levels of TNF-α and IL-6, but elevated levels of IL-1 compared to the control group (73). Research conducted on neutrophils in individuals with AN revealed diminished adhesion and lower bactericidal and cellular activities, resulting in heightened vulnerability to infections (74). An 8-year cohort study investigating the causes of death among individuals with AN revealed that 29% of deaths were attributed to infections, ranking second after deaths caused by AN or malnutrition (75). Sepsis is a critical infection-related illness (1), and the possible mechanisms by which AN leads to sepsis are currently focused on inflammatory factors, blood metabolites and gut microbiome (29, 76–83). Through a mediation MR analysis involving inflammatory factors, blood metabolites and gut microbiome as potential mediators, we discovered that blood N-formylmethionine levels and cystatin D levels accompanied by ketogluconate metabolism and N10-formyl-tetrahydrofolate biosynthesis in gut microbiome may mediate the causal association between AN and sepsis (84–86).

N-Formylmethionine is an amino acid found in bacteria and related eukaryotic organelles. It is a derivative of methionine that was initially identified during translation in bacteria, chloroplasts, and mitochondria. N-formylmethionine has historically been regarded as a marker of bacteria or bacteria-derived organelles (87). However, a recent study revealed an essential connection between human cellular N-formylmethionine and the susceptibility to age-related diseases in humans. Further investigation of the blood levels of N-formylmethionine confirmed its correlation with a higher likelihood of age-related diseases including renal disease and heart failure, as well as the risk of mortality (88). The precise function of N-formylmethionine in humans remains unclear, yet it exhibits potential as a biomarker for various age-related illnesses. Metabolic changes in AN result from chronic starvation and include disturbances in amino acids, lipids, and carbohydrates (89). Prior research has shown that individuals with AN frequently exhibit hyperaminoacidemia. Researchers have conducted targeted and untargeted metabolomics analyses of methionine levels in AN patient, with varying results showing enhanced, reduced, or stable levels of methionine (90–92). Formylmethionine deformylase can convert N-formylmethionine to methionine in prokaryotes (93). However, this conversion has not been observed in humans. Human mitochondria produce danger-associated molecular patterns (DAMPs) in the form of mitochondrial DNA and N-formyl-methionyl peptides when degrading. Mitochondrial DAMPs have been connected with dramatic disruption of metabolic balance, effective activation of innate immunity, and negative events in critically ill patients (94–96). The breakdown of the DAMP N-formylmethionyl peptide results in the release of N-formylmethionine into the bloodstream, indicating a significant disturbance in physiological homeostasis (97). A comprehensive metabolomic study analyzing critically ill ICU patients, including a sepsis cohort, showed that circulating N-formylmethionine promotes metabolic shifts and increased mortality, including incomplete oxidation of mitochondrial fatty acids, increased metabolism of branched-chain amino acids, and activation of the pentose phosphate pathway (98).

Cystatins are a group of protease inhibitors capable of inhibiting cysteine cathepsins, both inside and outside cells. Cystatin D belongs to type 2 cystatin and is encoded by the CST5 gene, which serves a regulatory role in the immunological response (99). Previous cross-sectional studies have discovered the existence of AN with the level of CST5 (100), and cystatin C has been found to possess greater diagnostic efficacy than creatinine in studies related to renal disease in patients with AN (101). A large Mendelian randomized study found a negative association between CST5 and various immune-related diseases such as gout, IgA nephropathy, primary sclerosing cholangitis, and sepsis (48). Research indicates that AN is correlated with unfavorable variations in the composition of the normal gut microbiota (102). According to a previous longitudinal study, individuals with AN possess substantially great abundances of Alistipes, Clostridiales, Christensenellaceae, and Ruminococcaceae and a lower abundance of Faecalibacterium, Agathobacter, Bacteroides, Blautia, and Lachnospira (84). A recent MR study revealed that AN was associated with increased levels of gut microbiome, including Alphaproteobacteria, Christensenellaceae, and Coriobacteriia (103). Variations in the gut microbiome make a person more prone to sepsis because they promote the growth of harmful bacteria in the gastrointestinal tract and trigger a robust proinflammatory response in the immune system (85). According to preliminary studies, individuals with less variety in their microbiome and a higher relative abundance of pathogenic gram-negative bacteria and enterococci are more susceptible to sepsis (86, 104, 105). Previous related studies have not further explored the relevant metabolic pathways in the gut microbiome. This study discovered that AN may increase the possibility of sepsis by increasing ketogluconate metabolism and N10-formyl-tetrahydrofolate biosynthesis in the gut microbiome, which may serve as a foundation for subsequent explorations for the specific metabolic patterns associated with gut microbiome alterations in AN-related sepsis.

In the present study, we investigated 91 inflammatory factors, 309 metabolite ratios, 1,091 blood metabolites, 205 gut microbiome pathways and 207 gut microbiome taxa and discovered that N-formylmethionine levels, cystatin D levels, ketogluconate metabolism and N10-formyl-tetrahydrofolate biosynthesis may possess mediating functions in the process of AN-sepsis. Currently, there is a dearth of studies on N-formylmethionine levels, cystatin D levels, ketogluconate metabolism and N10-formyl-tetrahydrofolate biosynthesis in patients with AN or sepsis. Further research is required to determine the role of the identified mediators in AN-sepsis.

The major strengths of this research are the adequate inclusion of multiple psychiatric disorders and the rigorous MR design. Data associated with psychiatric disorders were acquired from the PGC database, the most authoritative GWAS database for psychiatric disorders, to ensure the richness and credibility of the data. Causality between mental illness and sepsis was determined by MR analysis, and a bidirectional analysis was performed, ruling out reverse causality because of the negative effect of sepsis on mental illness. The causal relationship between mental illness and sepsis was further confirmed using meta-analysis and the MVMR method. Mediation MR analysis was employed for an in-depth investigation of the causal relationship.

Therefore, we believe that our findings are convincing and demonstrate a dependable causal explanatory impact. These discoveries may provide valuable insights for the prospective treatment of sepsis through further study of the mechanisms associated with mental illness. However, there are some limitations to our investigation. First, the applicability of our European population-based analysis to other populations cannot be determined. Although our findings demonstrate a causal relationship and discover a possible mediator between particular psychiatric disorders and sepsis, due to the limitations of GWAS data, the underlying mechanisms need to be further investigated in order to develop effective and feasible treatments for sepsis.

Mental illnesses and sepsis are significant health concerns today, and while there appears to be a causal link between them, it remains unclear. This study pioneers the use of the MR method to examine the relationship. By using genetic variations strongly related to mental disorders as instrumental variables, we assessed the causal connection between mental disorders and sepsis. Bidirectional MR, multivariable MR and meta-analysis were utilized for validation. Additionally, mediation MR helped us understand the roles of blood metabolites, inflammatory factors and gut microbiota in this relationship. These findings lay the groundwork for advancing the understanding and treatment of both mental illnesses and sepsis, with the ultimate goal of reducing incidence rates, mortality, and the global burden on health systems.

## 5 Conclusion

In summary, this research revealed that AN is independently associated with an increased likelihood of sepsis. Additionally, mediation MR analysis indicated that blood N-formylmethionine levels and cystatin D levels accompanied by ketogluconate metabolism and N10-formyl-tetrahydrofolate biosynthesis in gut microbiome may mediate the causal connection between AN and sepsis.

## Data availability statement

The original contributions presented in the study are included in the article/Supplementary material, further inquiries can be directed to the corresponding author.

## Ethics statement

Ethical approval was not required for the study involving humans in accordance with the local legislation and institutional requirements. Written informed consent to participate in this study was not required from the participants or the participants' legal guardians/next of kin in accordance with the national legislation and the institutional requirements.

## Author contributions

YH: Formal analysis, Writing – original draft. ZX: Methodology, Writing – original draft. PH: Investigation, Writing – original draft. WH: Investigation, Visualization, Writing – review & editing. MZ: Visualization, Writing – review & editing. DZ: Visualization, Writing – review & editing. GT: Supervision, Writing – review & editing.

## Footnotes

^1^ https://gwas.mrcieu.ac.uk/
