# Supplementary figures and images for: Association of mental disorders with sepsis: a bidirectional Mendelian randomization study
Source: Front Public Health. 2024 May 17;12:1327315. doi: 10.3389/fpubh.2024.1327315 (PMC11140049; doi:10.3389/fpubh.2024.1327315)

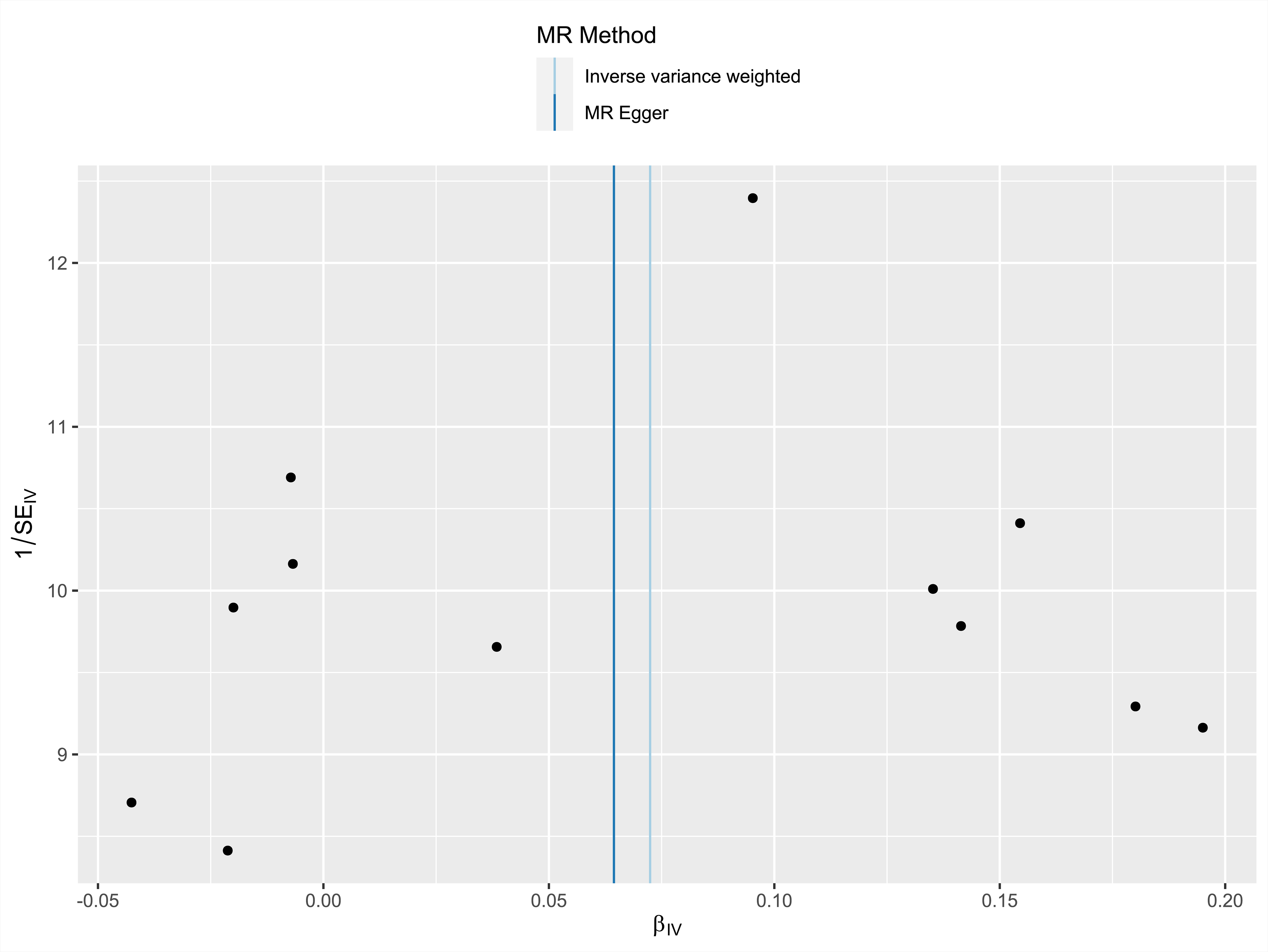

Supplement: Supplementary file 2 [file Image_1.TIF]
